# Navigating Comics: An Empirical and Theoretical Approach to Strategies of Reading Comic Page Layouts

**DOI:** 10.3389/fpsyg.2013.00186

**Published:** 2013-04-18

**Authors:** Neil Cohn

**Affiliations:** ^1^Center for Research in Language, University of California San DiegoLa Jolla, CA, USA

**Keywords:** comics, page layout, spatial representation, reading order, writing systems

## Abstract

Like the sequence of words in written language, comic book page layouts direct images into a deliberate reading sequence. Conventional wisdom would expect that comic panels follow the order of text: left-to-right and down – a “Z-path” – though several layouts can violate this order, such as Gestalt groupings of panels that deny a Z-path of reading. To examine how layouts pressure readers to choose pathways deviating from the Z-path, we presented participants with comic pages empty of content, and asked them to number the panels in the order they would read them. Participants frequently used strategies departing from both the traditional Z-path and Gestalt groupings. These preferences reveal a system of constraints that organizes panels into hierarchic constituents, guiding readers through comic page layouts.

## Introduction

Many readers of comic books have had the unusual experience of meeting someone who is confused by the order of the images on a comic page. While most literate readers are familiar with the left-to-right and downward reading orders used in written language – the “Z-path” – those who do not read comics sometimes become confused when pages depart from the stereotypical grid layout. To comic readers, this confusion seems baffling – isn’t the order of images obvious? While the sequential aspect of comics has often been emphasized as one of its defining features (e.g., McCloud, 1993), research has mostly examined how sequence conveys *meaning* (e.g., McCloud, 1993; Saraceni, 2001; Cohn et al., 2012; Cohn, 2013), with little attention paid to the overall page layout outside its impact on this comprehension (e.g., Barber, 2002; Cohn, 2003). Thus far, no research has broached the question of *how* readers create this deliberate sequence out of the unconstrained spatial array of analog visual information – the *external compositional structure* (ECS) of comic pages – and to what extent experience might play a role in guiding these decisions.

**Figure 1:**
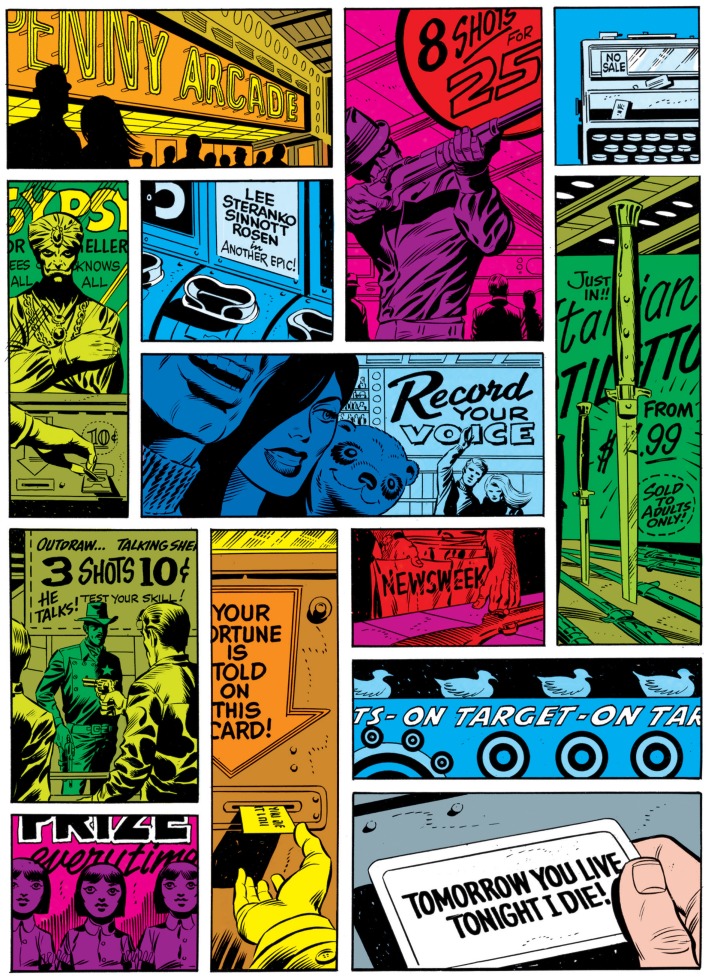
**Comic page by Jim Steranko that flouts a conventional linear path of reading (reprinted in Steranko, 2002)**. Image © 2002 Marvel Comics.

Scholarship on comics has mostly focused on the relationship of layout to the content of the images. Several authors have proposed taxonomies of layout types based on how they relate to the content of the narrative (Peeters, 1991/1998; Groensteen, 2007; Caldwell, 2012). For example, does the page serve a decorative function or does it use a standard conventional layout, such as a grid? Other conflations of layout and meaning have incorporated aspects of page layout directly into the comprehension of sequential images (Barber, 2002; Drucker, 2008). For example, Barber (2002) argued that comic pages are understood holistically through integration of the content of all panels on a page. Barber claims this idea is exemplified by a page from comic author Jim Steranko, depicted in Figure 1. This page allows for no contiguous columns or rows of panels, and the colors of panels imply perceptual groupings between non-adjacent panels, thereby making a linear reading order difficult.

While layout and content likely interface in important ways, they are ultimately independent structures. The same sequence of images can be arranged in multiple ways without impairing meaning. For instance, four-panel comic strips in newspapers commonly appear horizontally, vertically, or stacked in a 2 × 2 grid. In all cases, the content remains the same, while the layout changes. Granted, change in the *order* of panels would result in different meaning, but this still requires a method to explain *why* people read in the sequence they do. Navigational strategies cannot wholly rely on content, since once a panel is reached, readers would need to fully engage all possible choices of panels before choosing which one is next. This would place too much burden on the reading process, not to mention working memory. The smooth motions found in eye-tracking studies of comic pages seem to support that expert readers do not explore all options before moving from one panel to the next (Nakazawa, 2002). Moreover, while alterations in panel layouts affect eye movements, they do not appear to significantly impact reading comprehension (Omori et al., 2004).

Thus, a more basic question needs to be addressed: how do people know how to navigate through page layouts? Many factors likely contribute to how a reader might traverse through a comic page. These may include aspects of content, such as color of panels (as in the Steranko page), composition within a panel, character positioning or eye-gaze, or elements breaking the borders of panels (such as figures or speech balloons). Additional factors may fall outside the realm of content entirely, such as particular ways in which panels are arranged relative to each other (discussed below).

While research on comic page layout has not yet been undertaken, various studies have examined how readers engage other media, such as newspaper pages or websites. Experiments using eye-tracking have generally shown that readers scan these pages broadly, then focus on particular *entry-points* before they begin focused reading, usually with attention captured by images and larger items (e.g., Garcia et al., 1991; Kress and van Leeuwen, 1996; Homqvist et al., 2003; Holsanova et al., 2006). Unlike comics though, these media present readers with an unconstrained array of numerous types of information – images, headlines, advertisements, and articles. In contrast, the combination of text and image in comic panels are usually intended for only a single directed stream of reading – more similar to the stream of text in written language. Here, order has become conventionalized into different directional streams. English segments text into horizontal rows, and runs left-to-right and downward (a Z-path), while Japanese uses the opposite order, organizing text into vertical columns to read downward then right-to-left.

Despite the fact that comic page layouts often diverge from the uniform lines of text, most assume that comic pages follow the path of the culture’s written language (e.g., Bongco, 2000; Duncan, 2000; McCloud, 2000). Indeed, studies suggest that the orientation of a person’s writing system can impact other facets of perception. For example, left-to-right writing systems bias participants to prefer that directional ordering for depicting temporal relationships (Tversky et al., 1991; Chan and Bergen, 2005), for assigning semantic agency to objects (Maass and Russo, 2003; Dobel et al., 2007), for perceptually scanning arrays (Padakannaya et al., 2002), and for drawing pictures (Vaid et al., 2002), while the opposite has been found in right-to-left reading cultures. Given this, we might expect the left-to-right reading orientation to engender the use of a Z-path as well.

**Figure 2:**
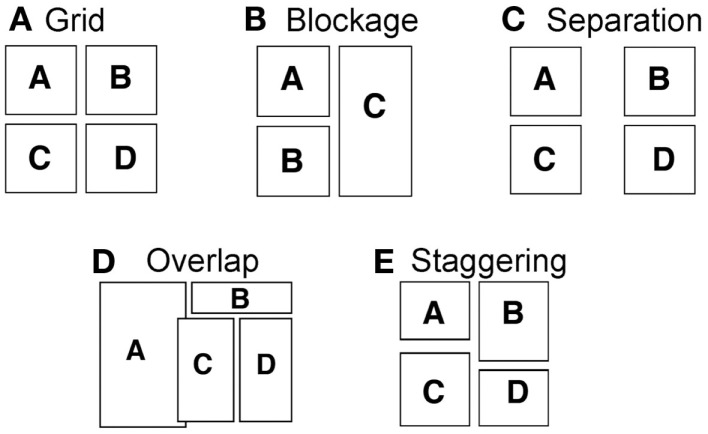
**Manipulations of comic page layouts. (A)** Canonical grid layout stereotypically read in a “Z-path.” **(B)** Layout where a horizontal panel “blocks” the creation of a row of panels. **(C)** Layout where panels are separated by a wide space. **(D)** Layout where panels overlap each other. **(E)** Layout where panels are staggered to no longer retain a contiguous gutter.

A Z-path certainly makes sense for a grid organization of panels (Figure 2A), which is most similar to the rows of text, but layouts may depart from or manipulate a straightforward grid in several ways. The borders of panels may become angled or eliminated, or panels might take strange sizes or shapes. These manipulations are relatively superficial though, since they may not necessarily force a reader to question how to order the panels. More challenging manipulations might vary the *proximity* of panels with each other, either through *separation* of the panels from each other (Figure 2C) or *overlapping* panels on top of each other (Figure 2D). This dimension of proximity has been well established as an organizational principle by Gestalt psychologists (e.g., Wertheimer, 1923) who showed that people perceptually group items that are nearest to each other. Would such groupings be preferred if they flout the Z-path?

Other orientations between panels create different challenges. On a small scale, panels may be *staggered* (Figure 2E) which might lead readers to question the Z-path because the horizontal gutter no longer runs continuously across panels to form a row. The most extreme manipulation of this type occurs when a whole panel “blocks” the horizontal gutter entirely. *Blockage* occurs when panels are stacked vertically next to a panel that runs the distance of the vertical panels. As in Figure 2B, following the Z-path causes panel C to be ordered before panel B. Thus, any subsequent panel (like B) might require backtracking in the opposite direction to the Z-path, thereby passing over the bottom part of panel C. An alternate order would move vertically before horizontally, where panel C “blocks” the Z-path, forcing movement vertically from A to B then horizontally to C. Do readers prefer to follow blockage or the Z-path in these situations?

In order to investigate how these factors influence the navigation of page layouts, we designed an experiment that presented participants with 12 comic pages with empty panels (i.e., with no imagistic content – only panel borders). Participants numbered panels in the order that they would read them, and we examined their ordering preferences for various manipulations to layout. Each of these manipulations will be described one at a time, followed by an analysis of the results. Finally, a theoretical model for the navigation of page layouts integrates this empirical data.

## Materials and Methods

### Participants

One hundred forty-five individuals (98 male, 47 female, mean age: 25.4) from the 2004 Comic-Con International comic book convention in San Diego, CA, USA participated in the study. Attendees were asked at random to participate in the experiment at a convention booth, and the sample reflects only those who volunteered. All participants gave their informed written consent and received a novelty sticker as compensation.

Prior to the experiment, all participants completed a questionnaire assessing their comic reading and drawing habits both in the present and childhood on a 1–4 scale (1 = Never, 4 = Always). This questionnaire also asked how often they read Japanese comics (*manga*), which often retain their native right-to-left reading order even in English translations. Table 1 summarizes participants’ background expertise.

**Table 1:** **Number of participants belonging to varying levels of comic reading expertise**.

	Never	Rarely	Sometimes	Often
Childhood comic reading	13	34	44	54
Current comic reading	7	36	54	47
Japanese manga reading	50	42	32	21

Note that one participant did not provide an answer for “current comic reading.”

### Stimuli

**Figure 3:**
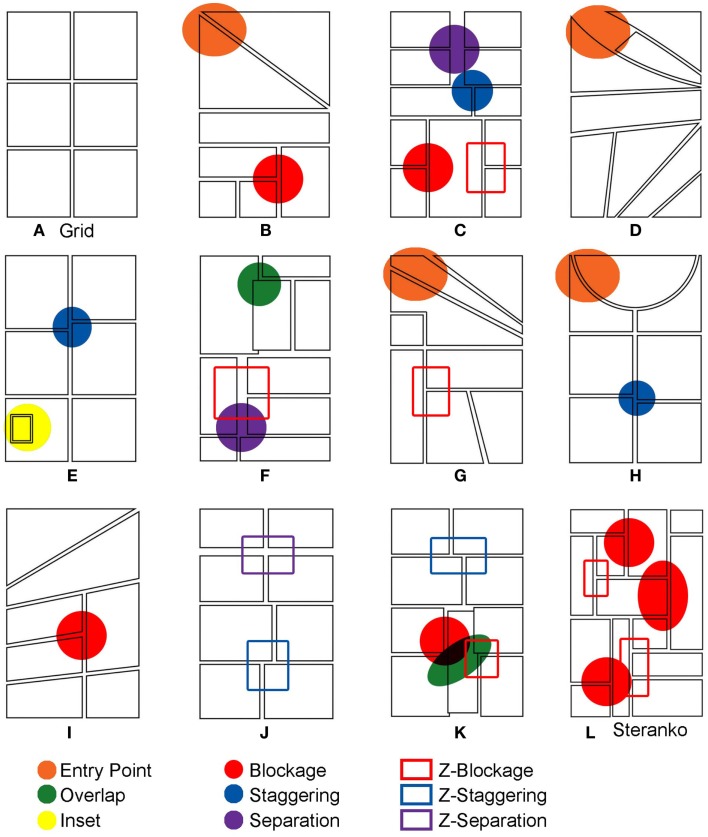
**All stimuli pages with notations of the various ECS manipulations**.

Each booklet consisted of 12 “comic pages” where the panels were empty of content (all stimuli pages found in Figure 3). All pages were created specifically for this experiment, except for one imitating the Steranko page discussed in the introduction (Figure 3L). Eleven of these page layouts tested various phenomena against the assumed Z-path, as well as additional properties that factor into the reading of pages. Each of these manipulations will be discussed individually below. Pages often featured several manipulations, though our analysis examined the adherence to the Z-path for isolated segments of the layouts vs. deviations using other orderings. Any reading order that was confounded by interaction with other parts of the layout was judged as a “non-Z-path” order. Results and discussion for each manipulation are discussed individually.

One page featured a 2 × 3 panel grid where columns and rows were clearly defined. While technically ambiguous for its reading order, we predicted this page to be ordered in the Z-path because of a grid’s similarity to text and its status as the most basic and conventional type of comic page layout. As such, this page was used as a control to be compared with other reading strategies. The additional manipulations of pages were embedded across panels to varying frequencies (as labeled in Figure 3). Each manipulation is described below.

#### Blockage

Seven instances of blockage occurred throughout the booklets (Figures 3B,C,I,K,L). In some cases, blockage featured a simple three-panel interaction (two vertical panels, one horizontal), while others featured more complex combinations. However, all used the same general interaction with a long vertical “Blocking Panel” that “blocks” the path of two or more rows of panels. It should be noted that three instances of blockage came from the Steranko page (Figure 3L). Detailed analyses of this page are provided in supplementary material available online at: http://www.visuallanguagelab.com/essays/ECS_Supplement.pdf

#### Separation

Two instances of separation were tested across the experimental pages (Figures 3C,F). In both cases, a large gap separated panels and grouped them in a way counter to the Z-path that sponsored a vertical path of reading.

#### Overlap

Two instances of overlap appeared throughout the booklets (Figures 3F,K). In the case of the second instance (Figure 3K), the overlap across three panels could reinforce both a blockage path (guiding the reader from the bottom left panel diagonally upward) or a Z-path (guiding the reader horizontally, then down to the diagonal left).

#### Staggering

Each booklet contained three instances of staggering (Figures 3C,E,H). In all cases, the borders between panels were staggered so that a continuation of the gutter moved vertically against the Z-path, instead of horizontally with the Z-path.

#### Insets

One page featured a single inset panel inside of a larger dominant panel (Figure 3E).

#### Entry-point

An additional manipulation looked beyond how participants navigated through clusters of panels, and focused on participants’ preferences when entering a page with no “entry-point” in the upper left corner of the page. Four of the experimental comic pages had no clearly defined panel in the upper left corner of the page (Figures 3B,D,G,H). These pages either divided this space between two or more panels or omitted a panel in this location altogether. Reading preferences for these pages were contrasted with the frequency that participants began a page with the upper left panel in the remaining eight experimental pages. This “standard” order was used as a control to compare against the experimental manipulations.

#### Fillers

In addition to these panel manipulations that challenge the Z-path, “fillers” introduced aspects of layouts that appeared similar to the challenging organizations, yet did not violate the Z-path. For example, “Z-blockage” manipulations horizontally flip a “blockage” layout by using a long vertical panel to the left of horizontally stacked panels. This order appears similar to blockage (as its mirror), but does not challenge the Z-path of reading. Similarly, “Z-staggering” and “Z-separation” used these manipulations of layout to reinforce a Z-path of reading. These fillers were included to give variation to the experimental page layouts and give the appearance of complexity while not challenging the Z-path (see Figure 3).

### Procedure

Each participant received a booklet with the 12 experimental layouts. All participants saw the same page layouts, randomly ordered in four different booklet sets. Participants were instructed to number the panels in the order that they would read them, and to treat all pages as independent (i.e., that there were no “two page spreads”). Additionally, participants were told that there were no “right or wrong” answers, and to follow their own intuitions. Participants filled out the booklets with no time restrictions, though most averaged between 5 and 10 min.

### Data analysis

We first wanted to know how often participants used the Z-path given a particular manipulation in the layout. For each manipulation, we calculated the mean frequencies that a participant followed the Z-path by collapsing across all instances of a given manipulation. An Independent Samples *t*-test compared these means with those of a control (i.e., the means for following the Z-path in the grid). We next wanted to know which strategy each participant chose more for each manipulation: the Z-path or an alternate route. For a given manipulation, we calculated whether each participant used the non-Z-path over 50% of the time (a value of “1”) or used the Z-path (“0”). Across all participants, a sign test was used to analyze which strategy was more frequently used overall.

Finally, we wanted to know how particular strategies of navigation were influenced by participants’ background experience with comics. Participants were grouped based on their self-assessed levels of expertise (i.e., Never read comics vs. Sometimes vs. Often vs. Always) in a variety of fields (frequency reading/drawing comics or Japanese manga, as an adult or child). Mean frequencies of navigational strategies were compared across participants’ expertise ratings using a one way analysis of variance (ANOVA) that set levels of expertise as a between-subjects factor. Significant interactions were followed up using *post hoc* Independent Samples *t*-tests.

## Results and Discussion

### Grid

The most conventional layout of a comic page is a grid. Within our booklets, one page featured a 2 × 3 panel grid (Figure 3A). Technically, this page could be ordered in any number of ways (Z-path, up and down, snaking in a reverse “S” shape, etc.), though the canonical order would follow the Z-path of the standard reading order of the English writing system. Indeed, participants did choose the Z-path almost all the time (*M* = 0.941, SD = 0.172). The only instances of departure from the Z-path were participants who ordered the panels in vertical columns. Comparison of participants’ frequencies of using the Z-path with frequencies of vertical orders showed that this preference was clearly significant, *z* = −10.796, *p* < 0.001. Because it is the standard reading order in the most conventional layout, the mean preferences for the Z-path of the grid were subsequently used as the control for analysis of other manipulations to layouts. We now turn to discussing more complex manipulations of page layouts.

### Blockage

We first examined page layout segments using blockage, as in Figure 2B. Blockage has often been cited as “problematic” by comic creators and inexperienced comic readers (Abel and Madden, 2008). Some evidence supports this claim. In a study looking at eye movements when readers viewed comic pages, Omori et al. (2004) found that readers frequently skip over the vertically aligned “B” panels (as in Figure 2B) when presented with blockage situations, and that when modified to a horizontal path, skipping of this panel decreased dramatically. This would indicate that participants would prefer the horizontal Z-path when presented with these situations.

Orders followed the Z-path if a horizontal path progressed from the upper row of panels to the Blocking Panel prior to moving down to lower panels (i.e., the “ACB” path in Figure 2B). Orders that followed the “blockage path” moved vertically to complete the rows of horizontal panels prior to numbering the large Blocking Panel (i.e., the “ABC” path in Figure 2B).

**Figure 4:**
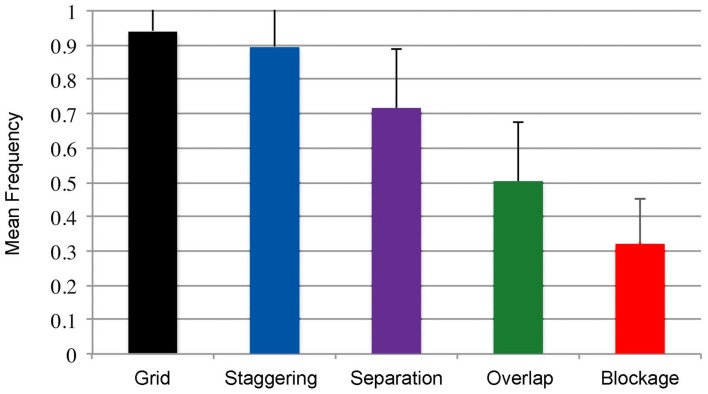
**Frequency of using the Z-path under various manipulations**.

Blockage clearly influenced participants to depart from the Z-path. Participants ordered panels in the Z-path infrequently in blockage scenarios (means are summarized in Figure 4), which was significantly less than the frequency of Z-path usage in the control grid, *t*(144) = −22.79, *p* < 0.001. Closer analysis showed that, when faced with a blockage situation, more participants preferred to move to a vertical panel (91 of 145) than moving to a horizontally adjacent panel (40 of 145). These data show that, when posed with a blockage situation, participants chose a route differing from the Z-path over twice as often as when they followed it, a clearly significant difference, *z* = −4.37, *p* < 0.001.

**Figure 5:**
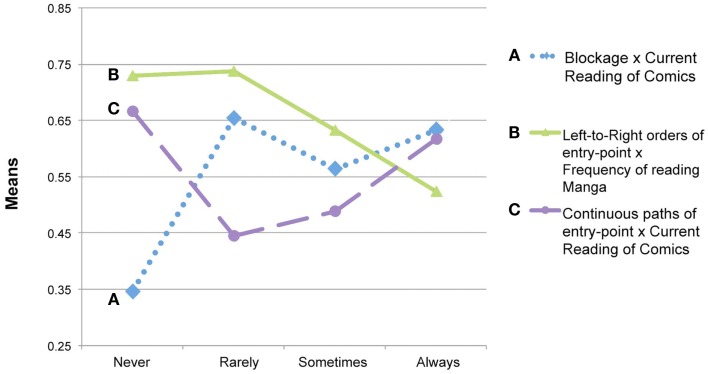
**Influence of background expertise on various strategies of navigating page layouts**.

Usage of the blockage path appeared to be affected by the frequency participants currently read comics. The blockage path increased along with participants levels of reading frequency (summarized in Figure 5A) compared to those who do not read comics at all, *F*(3,140) = 2.964, *p* < 0.05. Follow up analyses revealed that participants who never read comics had fewer frequencies using the blockage path than those of any other group to a significant or trending degree (all *t* > 2.0, all *p* < 0.054), while those with any habits of reading comics did not differ from each other (all *t* < 1.58, all *p* > 0.126).

These results show that, when presented with blockage situations, comic readers clearly preferred using the blockage path of navigating through comic panels over the Z-path. These results contrast with the findings of Omori et al. (2004), where readers’ eyes often followed the Z-path of reading. This discrepancy in results may be attributable to comic reading expertise. The data on comic reading expertise indicate that people who read comics at least to a moderate amount have a greater tendency to prefer the blockage path to those who do not read comics at all. Overall, that novice comic readers would prefer the Z-path makes sense: since they are unfamiliar with blockage scenarios, they revert to the comfortable Z-path inherited from written text. Indeed, additional eye-tracking research has shown that the saccades of a novice reader were far more erratic and less directed than an expert reader (Nakazawa, 2002). Thus, the results in Omori et al.’s (2004) study could have reflected participants with less experience in reading comics, though such expertise measures are not reported.

### Separation

Page layouts may also manipulate constraints of Gestalt principles of grouping (e.g., Wertheimer, 1923) such as proximity. As discussed, Gestalt psychology has long shown that people perceptually group items that are nearest to each other. Thus, in the *separation* condition, panels were grouped in ways that might violate the Z-path by extending the space between columns of panels, as in Figure 2C. Here, to maintain the Z-path (i.e., A–B), participants would need to jump over a large gap between panels, instead of following the most closely grouped panels (i.e., A–C). If participants are sensitive to the Gestalt preference for closely grouped panels, they will depart from the Z-path to order panels that are nearest together. If the Z-path remains dominant, participants should be unaffected by the groupings created by separating panels from each other. Thus, a separation path followed the Z-path if orders crossed the separation to maintain a left-to-right path, while orders that did not follow this path, moving to the panels grouped closest together, followed the separation path.

While participants followed the Z-path for separation situations in high proportions (*M* = 0.717, SD = 0.338, see Figure 4), this frequency of ordering panels still significantly differed from that of the control grid, *t*(144) = 6.758, *p* < 0.001. Overall, when faced with separation conditions, more participants chose a panel along the Z-path (84 of 145) than an alternative path. This difference was significant, *z* = −6.97, *p* < 0.001.

These results indicate that separation of panels did impact participants’ preferences for navigational order, though it was not influential enough for full rejection of the Z-path. Participants chose the Z-path almost three times more than they chose the Gestalt groupings created by separation. Nevertheless, separation does play a statistically significant role in altering the strategies of navigation away from the Z-path when compared to the control condition. Further analysis of these contrary results will be addressed in the Section “General Discussion.”

### Overlap

The Z-path could also be flouted by the reverse proximity relationship to separation: when panels are close together. This happens to the extreme when panels *overlap* each other, and the borders of one panel lie on top of another. Like separation, this manipulation can flout the Z-path, as in Figure 2D. The proximal relations between overlapping panels (i.e., A–C), force a reader to choose to either move upwards (to B) or laterally (to C) and then upwards. This would force them to skip back over panels they have already read. Alternatively, following the Z-path (i.e., A–B) would allow panels to be read in a familiar left-to-right and down fashion, but would require ignoring the clear grouping created by the overlap of panels (i.e., A–C). If readers prefer the Z-path, they should ignore the Gestalt effects of overlap. However, if the Z-path does not guide their preferences, the Overlapping Panel would be ordered prior to those guided by the left-to-right and down order.

Overlapping Panels did influence the navigation order. Roughly half of all orders followed the Z-path in these situations (*M* = 0.50, SD = 0.341, see Figure 4), which differed from the preferences of the control grid, *t*(144) = 13.828, *p* < 0.001. Additionally, when presented with an overlap scenario, more participants chose to follow the overlapping panel than the other choices available to a proportion trending in significance, *z* = −1.74, *p* = 0.081.

While overlap significantly influenced the grand mean for ordering of panels, these results were largely influenced by the nature of the path created by the overlap. Analysis at each instance of overlap revealed that preferences for departing from the Z-path differed between the two stimuli. While the overlap in Figure 3F clearly showed a preference for the Z-path, only rarely departing from it (*M* = 0.17, SD = 0.37), the overlap in Figure 3K did not, instead directing a preferred order following a blockage path (*M* = 0.63, SD = 0.48), which was a significant difference *t*(144) = −10.203, *p* < 0.001. In Figure 3F more participants chose the Z-path than other strategies (105 of 145), *z* = −7.04, *p* < 0.001. However, more participants dominantly followed the overlap in Figure 3K into a blockage path (92 of 145), *z* = −4.23, *p* < 0.001. Indeed, a blockage condition without overlap used this same layout, where the blockage path was chosen nearly 75% of the time. This indicates that preferences for following overlap in this stimulus had less to do with the influence of the overlapping panel, and more with other navigation strategies, such as blockage. Overall, these results imply that overlap on its own does not provide a sufficient influence to dramatically alter the preference for the Z-path.

### Staggering

Another type of Gestalt constraint has less to do with proximity, but more to do with the continuation of a common flow. This can be manipulated in comic layouts by altering the size of panels so that their borders do not line up cleanly to create a smooth row or column. Thus, *staggering* panels offsets the rows or columns so that the flow of panels does not create a clear grid, as in Figure 2E. Following the staggered panels orders panels along straight gutters (i.e., A–C) instead of following the Z-path (i.e., A–B). Reading downward would avoid conflict with the offset created by the staggered borders. Thus, like the effects of blockage, staggering could force navigation away from the Z-path by following the flow of the clearly defined gutters. Thus, experimentally, we would predict that if participants prefer the Z-path, the staggering of panel borders should have little effect on their maintenance of a left-to-right order.

Orders were deemed as following the Z-path if they maintained the left-to-right ordering that did not choose panels along the vertical dimension. Staggering barely impacted the ordering of the Z-path. The Z-path order dominated all preferences in staggering scenarios (*M* = 0.90, SD = 0.23, see Figure 4), though this trended toward differing with the frequencies of the Z-path in the control grid, *t*(144) = 1.788, *p* = 0.076. Additionally, far more participants preferred the Z-path (131 of 145) to other strategies, *z* = −10.59, *p* < 0.001.

Staggering had the least impact on participants to depart from the Z-path. These results are curious because staggering is very similar to blockage: they differ only in the degree to which the stagger meets the Blocking Panel. Though blockage had a huge impact on orders of panels, staggering did not. Given this similarity, the question remains at what point does staggering yield the same effects as blockage? In the examples used, the staggering only subtly varied the distance that divided the continuous flow of the lateral gutter. Would a more dramatic stagger yield a stronger blockage effect? Will staggering always overcome blockage as long as there is more than one panel stacked as “blockers”? Future studies could address these concerns (see the General Discussion).

### Insets

Further aspects of navigating comic pages do not involve the Z-path. For example, one panel can enclose another as an “inset” panel. Inset panels feature one “enclosed” panel embedded within another “dominant” panel. The question here is which panel readers prefer to be ordered first: the enclosed or dominant panels?

Analysis of the inset scenario showed that 60% of the time the outer “dominant” panel was ordered before the enclosed panel (84 of 145), a significant difference, *z* = −2.47, *p* < 0.05. In many ways this order makes sense, since the reader engages the borders of the outer panel first, after which they progress to the inner panel. Coming to the inner panel first would make the reader skip over the outer panel when it is initially approached.

### Entry-point

Finally, navigating page layouts also involves where people prefer to begin reading a page. Eye-tracking studies on reading strategies in newspaper or website pages have shown that attention often becomes directed to larger items or more colorful elements (Holmqvist and Wartenberg, 2005; Holsanova et al., 2006; Homqvist et al., 2003). Pages like these are often scanned, with certain segments acting as *entry-points* for directed reading of text, such as dominant photos or particular snippets of text (Garcia et al., 1991), though researchers disagree about whether leftward or rightward elements attract more attention (Arnheim, 1974; Garcia et al., 1991; Holsanova et al., 2006; Homqvist et al., 2003; Kress and van Leeuwen, 1996). However, unlike these unconstrained spatial arrays, comic pages have an intended reading order, and are recognized as conveying explicit linear streams of information, similar to writing systems. Thus, conventional wisdom would say that readers of left-to-right writing systems would prefer to start in the upper left-hand corner of a page, while readers of right-to-left writing systems would prefer the upper right corner. Research has supported that the order of a person’s writing system affects other aspects of their spatial cognition. For example, readers of left-to-right writing systems are better able to recall elements in the upper left quadrant of an array, while readers of right-to-left systems better remember upper right quadrants (Chan and Bergen, 2005). Such results would imply that similar preferences would be maintained for comic pages.

Thus, if comic panels use the same intuitions as text, participants should prefer to begin pages in the upper left. To test this, participants were presented with various pages where the upper left corner lacked a clearly defined panel. We asked whether such examples pose problems for participants, and whether consistent strategies were employed to handle such irregularity.

Analysis of experimental pages looked at several factors. First, we examined where the first chosen panel was located relative to upper left corner space. In cases where this space was divided into two parts diagonally, panels were either deemed as left/bottom vs. right/top. Second, we examined the nature of the path involved in ordering panels surrounding these situations. Paths ordered with a left-to-right path followed an overall left-to-right direction across panels. This left-to-right order also was a bottom-to-top order given in the diagonal relations of the panels. Right-to-left orders followed the opposite directionality. Finally, continuous paths were compared with broken paths. Continuous paths successively ordered adjacent panels. Broken paths jumped between non-adjacent panels, thereby skipping over panels in-between.

As would be expected from a Z-path, participants strongly preferred starting comic pages in the upper left corner. This preference was so great that one participant actually numbered “1” into the empty space where an entry panel normally would appear (perhaps considering this as a “borderless panel”). In pages with a panel in the upper left corner, that panel almost always began the page (*M* = 0.96, SD = 0.13). By comparison, when no panel was clearly defined in this position, the preference for choosing the *leftmost* panel dropped dramatically (*M* = 0.60, SD = 0.20), a significant difference, *t*(144) = 21.26, *p* < 0.001.

This leftmost preference extended to orders of panels as well. The left-to-right order was far preferred to a rightward order. Significantly more participants chose a left-to-right/bottom-to-top order (93 of 145) than chose alternative orders like right-to-left (21 of 145), *z* = −6.65, *p* < 0.001. Reading frequency of Japanese manga had a significant influence on the left-to-right order of panels, *F*(3,141) = 3.866, *p* < 0.05. A high frequency of reading Japanese manga decreased the likelihood of using a left-to-right ordering of panels, as depicted in Figure 5B. Follow up analyses revealed that participants who often read manga had significantly fewer frequencies of left-to-right orders than those of people who never or rarely read manga (all *t* > 2.83, all *p* < 0.01). All other contrasts between groups were not significantly different from each other (all *t* < 1.69, all *p* > 0.096).

The interaction of left-to-right motion with frequency of reading Japanese manga may support that reading of different page layouts alters one’s navigational preferences. Participants who read more manga had a reduced likelihood of using a left-to-right motion, consistent with their reading habits: manga pages, even when translated into English, often maintain the original right-to-left orders. Despite these right-to-left orders in the entry-points of these pages, panels in the whole pages were ordered left-to-right. Thus, the effects of manga reading seem to influence only where to begin reading a difficult page. These results are consistent with evidence that readers of writing systems with left-to-right paths attend to different quadrants of an array than those of right-to-left systems (Chan and Bergen, 2005). In this case, increased reading of manga pages that begin in the upper right lead participants to start pages in that area more often, even when that directionality does not persist through the rest of the page.

Age also positively correlated with left-to-right orders, *r*(142) = 0.253, *p* < 0.005, showing that preference for this order increased with older individuals. This could be attributed to several factors. For example, if it were the case that a higher proportion of younger readers read manga, they might rely more on the left-to-right orders. However, age correlated significantly with manga reading, indicating that this was not the case. An additional explanation may be that newer comics – those read more by younger readers – may use more complex layouts than older works familiar to older readers. If this is true, newer types of layouts might familiarize younger readers to alternative strategies, while older readers retain the left-to-right orders common to older comics. While it is generally accepted that mainstream American superhero comics have introduced more complexity to page layouts than their older or Indy genre counterparts, such a claim could be substantiated with corpus analyses.

A Gestalt constraint of continuity further influenced preference of entry-point, as participants chose continuous paths over broken ones. In pages with more than two panels in the upper left corner, more participants preferred continuous paths (88 of 145) over broken paths (48 of 145), *z* = −3.34, *p* < 0.005, even when they departed from the left-to-right path. As depicted in Figure 5C, continuous paths seem to have been most preferred by participants with the polar habits of frequent and infrequent habits of reading comics, *F*(3,140) = 5.12, *p* < 0.005. Participants who never or always read comics used continuous paths at frequencies significant or trending to be greater than those used by those who read comics only rarely or sometimes (all *t* > 1.95, *p* < 0.056). No difference was found between those who read comics never/always or rarely/sometimes (all *t* < 0.594, *p* > 0.423).

## General Discussion

Overall, these experiments showed that certain manipulations of a page layout push readers to flout the Z-path inherited from the reading of text. In particular, blockage and separation had the most impact on pushing readers to follow Gestalt groupings instead of the Z-path, while overlap and staggering had little effect. Additionally, several aspects of background reading habits influenced the frequencies at which individuals navigated comics, indicating that a degree of expertise can influence the navigation of comics away from the Z-path.

These findings raise additional questions: how long does a Blocking Panel need to be to invoke blockage, since staggering alone created little effect? Why does separation have a significant impact on navigational path, even though the Z-path was still chosen more than any other strategy in these situations? Addressing these issues requires that the data from these experiments be assimilated into a broader analysis. The following section sketches out a preliminary theoretical model for the governing principles underlying the navigation (and creation) of comic page layouts. First, I will propose a general principle of navigation based on preference rules for selecting the path of reading. Second, these rules will be situated as part of a broader generative model for the architecture of ECS.

### Constraints on external compositional structure

The results of these experiments reveal an overarching strategy that expands on the basic principles of the Z-path. By and large, the rightward and downward direction of the Z-path was preferred: participants started in the upper left and progress to the lower right. However, beyond this, readers were sensitive to various aspects of panels’ relations to each other and the page borders. To negotiate these issues, I propose that a general strategy of “Assemblage” seeks *to build successive units of structure based on distance and coherence of composite shapes in as smooth a reading path as possible*. There are several *general preferences* that guide Assemblage:

1. Grouped areas > non-grouped areas
2. Smooth paths > broken paths
3. Do not jump over units
4. Do not leave gaps

**Figure 6:**
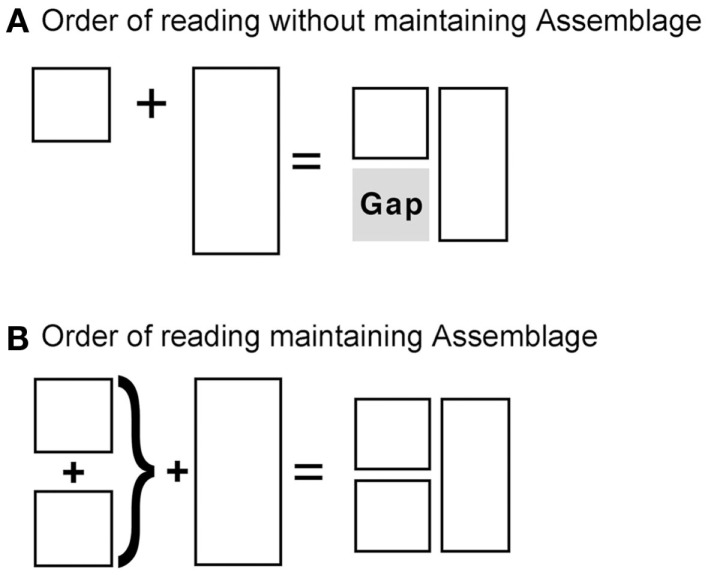
**Blockage preferences through Assemblage**.

Blockage can provide a good example. Assemblage would predict that readers follow blockage because a horizontal Z-path reading would leave a “gap” in the broader shape of the panels’ “additive space,” as in Figure 6A. In contrast, the preferred reading fills in the whole space in the most economical order possible through an additive process, as in Figure 6B. By moving vertically first, the combined space of the stacked panels equals that of the blocking panel to their right. Grouping these panels first ensures that no excess space remains at any point of the reading process, and that navigation follows a smooth path. This grouping relies on coherence of the lengths of the various panels’ borders. Two segments must guide this: the length of the top border, and the length of the inner vertical borders. In blockage, the vertical boundaries guide the navigation before the horizontal border. However, the horizontal boundary is retained as an overarching space required to be filled.

Assemblage acts as a general principle to the ECS of comic page layouts, while a more explicit set of preference rules more specifically direct the processes of navigation. Like the constraints placed on perception by Gestalt groupings, preference rules specify the “preferred” interpretation out of various possible structural interpretations, and have been used for describing both music cognition (e.g., Lerdahl and Jackendoff, 1982) and language (e.g., Jackendoff, 1983). In combination with the general principles of Assemblage, these “ECS Preference Rules” (ECSPR) sketch out the series of operations that occur when a reader is at one panel and looking to move to a subsequent panel. A navigational choice is determined by moving down the list: if a rule is met by all or no options, the next rule is invoked, down until a constraint is satisfied. Like the conflict that can occur between Gestalt constraints, these preferences rules operate in probabilistic ways, rather than algorithmically. Thus, these rules can come in conflict with each other and must be played off each other to determine the proper path. Depending upon the conditions, different rules may win out (for example, preferential ordering rules for blockage and the Z-path “won out” over the proximity of overlapping panels).

Below, each constraint will be discussed and then applied to examples. These descriptions provide an initial foray into describing the structures governing the navigation of page layouts. Future work can further investigate the probabilistic weights that guide these constraints further. It is also important to remember that these only comprise part of what is likely a larger family of navigational constraints also including the influence of the content of images within and across panels.

#### Entry constraints

Before actually navigating through various panels, readers must first find a starting panel. Thus, the first preference rules outline how a sequence is begun, when faced with an ambiguous page or display (as opposed to a scenario when the first panel is overtly provided, such as in digital comics that force the reading to begin at a particular panel).

***ECSPR E1: go to the top left corner***. The results of the analysis of entry-points suggest that readers consistently look for a panel in the top left corner of a page as the first preference for entry into a “canvas” (i.e., a page or screen). Note, cultural experience may alter the direction of this constraint (and all the preference rules), as indicated by right-to-left preferences from readers of Japanese manga.
***ECSPR E2: If no top left panel, go to either the (1) highest and/or (2) leftmost panel***. When no panel exists in the top left to satisfy the first constraint, this second constraint directs readers to balance either the panel that is most left on the page (so that a left-to-right reading motion is preserved) or the highest panel on the page (to preserve a smooth continuous path of reading motion).

#### Navigational constraints

**Figure 7:**
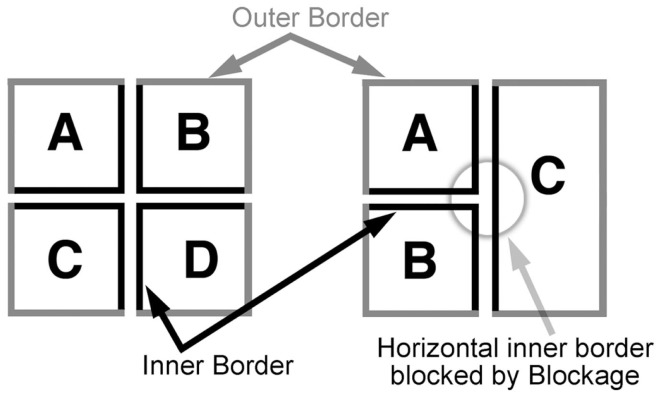
**Inner and outer borders of panels in a layout**.

Once an entry panel is established, navigational constraints specify how to move through that environment. The first two of these constraints involve following the inner and outer “borders” of panels (see Figure 7), while the others involve broader movements. “Border” in this case may be considered as an abstract. Panels often lack drawn borders, and it is unclear whether “borderless” panels would obey the same principles sensitive to borders or not.

***ECSPR 1: follow the outer border (Assemblage constraint 1)***. The first navigational choice seeks a contiguous edge of the outer-most borders of the situated panel and its immediate surrounding panels.
***ECSPR 2: follow the inner border (Assemblage constraint 2)***. If all available paths have contiguous outer borders, seek a contiguous border for the inner edges of a panel and its adjacent panels (see Figure 7). These rules following outer and inner borders reflect the desire of Assemblage to create groupings out of contiguous orders.
***ECSPR 3: move to the right (Z-path constraint 1)***. When either an outer or inner border can be followed, the first preferred motion goes to the right. In less expert comic readers, this preference may be elevated to the most preferred reading strategy, as acquired by the Z-path. In other words, these readers ignore the sensitivity to the borders and Assemblage, seeking only to satisfy a left-to-right reading path.
***ECSPR 4: move straight down (Z-path constraint 2)***. Given the previous constraints, if a rightward movement is unavailable, downward movement is next preferred.
***ECSPR 5: if nothing is to the right, go to the far left and down (Z-path constraint 3)***. Sometimes no panel is available to the right, such as at the end of a row of panels on a page, forcing the reader to move to the next tier down. This rule specifies the diagonal motion inherent in the Z-path. This rule comes into direct conflict with the previous rule as a separate type of downward movement. In those cases, the local context decides which rule wins out.
***ECSPR 6: go to the panel that has not been read yet***. The final rule provides a default for reading any panel that has not yet been read. As the terminating constraint, this rule cannot be overridden. In the case where panels are randomly scattered and “floating” on a page, the Z-path rules (3–5) may guide a reader in some semblance of order, while this last rule “sweeps up the remainders.”

As is labeled, the first two rules satisfy the constraints of Assemblage, while the remaining rules refine the process of the Z-path. Most likely, the default for readers is ECSPR 3–6 gained through the reading motions found in text, while experienced comic readers have acquired additional Assemblage constraints that take precedence over this default.

These rules can better be understood through examples, beginning with blockage. When presented with a layout like Figure 2B, ECSPR E1 is engaged first. Since a panel is present at the upper left corner, that constraint is satisfied anda reader first goes to panel A. From here, ECSPR 1 checks the outer borders: the contiguity of both outer borders is sustained (A–C and A–B), so this constraint alone cannot determine the path. Moving into ECSPR 2, panel C blocks the contiguity of the inner horizontal border, but the vertical inner border downwards to panel B is not blocked. Since panel C blocks rightward movement in ECSPR 3, ECSPR 4 initiates movement downward, and the constraints are satisfied reaching panel B.

In the case of a grid, as in Figure 2A, at panel A both ECSPR 1 and 2 are met, since both outer and inner borders are contiguous all the way through. This allows ECSPR 3 to be initiated since a rightward movement can reconcile the ambiguity, resulting in the common left-to-right motion of the Z-path. In situations with separation and staggering, we find competition between the Assemblage and Z-path constraints. Once at panel A, ECSPR 1 (and 2) can be satisfied by moving downward, invoking ECSPR 4. However, if the gap between panels is ignored as a constraining feature, the upper border from panel A can still be perceived as forming a contiguous line with panel B. This may invoke ECSPR 3 for rightward motion previous to ECSPR 4 coming to action. Such a result was observed in the experiment: while readers chose the Z-path (A–B) almost three times as much as the Gestalt grouping (A–C), the vertical path remained statistically significant. In this case, both pathways are acceptable, but it appears that the Z-path constraints “win out” over the Assemblage constraints more often.

A similar competition occurs for staggering, which trended toward influencing away from the Z-path. Here, the preference rules must weigh the influence of the discontinuous inner border forming a “continuous” gutter horizontally across panels against the continuity of an undisrupted vertical border. If the disruption in the border is perceived as significant – i.e., enough to warrant the blockage of that “row” – ECSPR 2 will guide the reading vertically instead of allowing the Z-path rules to push the motion rightward while following the outer borders. The results of this experiment showed that minor staggers to a horizontal row are not enough to push an order vertically, yet blockage was very effective in doing so. This issue may be influenced by the general Assemblage preference for forming grouped areas to non-grouped areas. So long as the layout allows a “constituent” to be formed horizontally, staggering may have less of an effect. One way that future experiments could test this would be to align a column of three equally sized panels next to another column of two equally sized panels, which would thus provide fully ambiguous staggered groupings (i.e., the two gutters between the three panels should be equally far from the gutter between the two panels). From here, slight variations in the size of panels in the two-panel column could vary whether its horizontal gutter aligns with the gutter between the first-second or second-third panels of the first column. Such a method could provide one way to assess how much stagger leads to blockage.

The ways in which preference rules can weigh different conflicting paths can be seen by the navigation of the unusual diagonal borders presented in the entry-point pages. For example, in Figure 3D, no panel occupies the upper left corner panel, making ECSPR E1 not satisfied. In this case, ECSPR E2 can select the leftmost panel as the entry-point. From here, ECSPR 3 guides a rightward movement, maintaining general Assemblage principle 3 for a continuous motion to create a grouping out of the whole tier of diagonal panels. This strategy was chosen the most often for this layout (67 of 143), but the next most chosen strategy moved from the topmost panel (which is more rightward) and then moved smoothly downward (45 of 143) (note: two participants skipped this layout). Both strategies satisfy ESPR E2 to start the page in different ways (i.e., left vs. top), which results in different directions of reading. However, once an entry-point is decided, the general Assemblage principle for a continuous motion is satisfied. Thus, in these cases, the overall desire for a continuous motion may provide a stronger influence that the precise entry-point.

The choice of ESPR E2 to choose a leftmost vs. topmost panel may also be influenced by the size of panels or surrounding context. For example, in the entry-point of Figure 3B, where a binary choice was offered, the leftmost panel was chosen far more than the top one (104 of 145). In contrast, readers chose the topmost panel in the seemingly binary choice in Figure 3H (114 of 145). However, in this case, the thin sliver of the leftmost panel is connected to a panel that forms a contiguous lower border with an adjacent panel. Here, participants seem to consider the “bottom” panels as forming their own “row” beneath the “row” of the topmost curving panel, thus enabling a Z-path of reading. This means that the thin strands of the bottom panels in this “top row” are insufficient on their own to motivate being read prior to the topmost panel. Creating rows in this way satisfies general Assemblage constraint 1 for the creation of a “grouped area.” This entry-point example and the examples of staggering show that the creation of grouped areas does not necessarily depend on contiguous gutters (see also the analysis of the Steranko page in supplementary material). This point of creating constituents will be returned to in the next section.

While these preference rules and general Assemblage constraints provide an initial foray into describing the governing principles of comic page navigation, it should be clear that these experimental results are not enough to fully articulate the precise balancing of these principles. There are two directions that need to be addressed in future works. First, this first study on ECS only examined these manipulations in broad strokes. Further studies can examine the precise probabilistic weights negotiated by the ECSPR to determine how one rule is chosen over another. For example, how discontinuous does a horizontal gutter need to be to create a blockage effect? How large a gap does a separation need to be? What physical features might motivate an entry-point to be at the leftmost vs. topmost panel? These constraints would deal with the direct panel-to-panel choices in the navigation of pages.

A second line of research must further address how the local constraints of the preference rules interact with more global principles offered by the Assemblage constraints. In several cases, the choices for local directions in page navigation were influenced by the overall interactions between panels on a page – sometimes by non-adjacent panels that happened to form a larger grouping. Here we find the balance between the analog nature of comic pages as visual-spatial arrays – presenting all the information at once – and the linear stream of panels creating a cohesive visual narrative. Further articulating the balance between these issues must be addressed more precisely in future studies.

### Embedding structures

The ECSPR and Assemblage constraints serve as principles that govern how a reader might navigate from panel to panel in a page layout. As described, one constraint on a preferred strategy in navigation is the creation of “grouped areas” over “non-grouped areas.” Such an overall constraint may be guided further by underlying structures that form hierarchically embedded constituents, which essentially define these “grouped areas.” It may be the case that the general constraints and preference rules function in order to build such hierarchic structures while a reader is navigating through a page layout. Conversely, these structures may motivate comic creators in their designs of page layouts, which are then “decoded” through a reader’s navigation. A simple model of these types of relations was proposed by Tanaka et al. (2007) for converting comic pages into an acceptable format for cell phone and PDA screens. Their approach divided pages into vertical and horizontal segments that recursively embed into each other. A similar approach will be presented here, with further elaboration.

**Figure 8:**
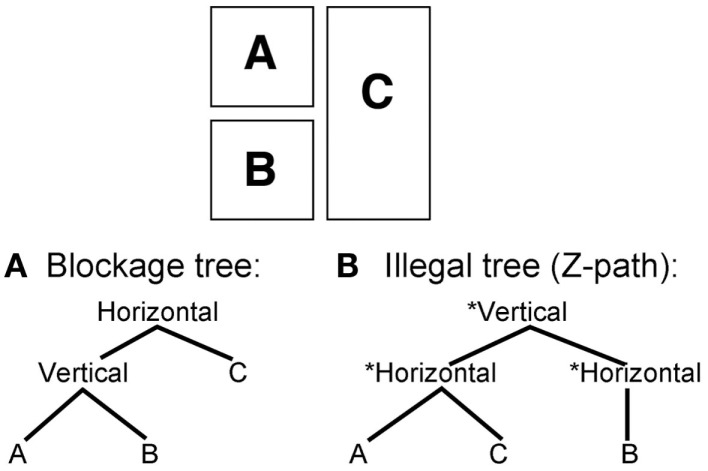
**Tree structures for the reading orders of blockage using (A) a strategy following Assemblage, or (B) an infelicitous Z-Path**.

Visual constituents are naturally created out of the Gestalt groupings of the navigational preference rules. Constituents become formed by vertical and horizontal groupings, which concatenate panels additively. For instance, Figure 8A depicts the Assemblage process that guides blockage, where a vertical composite unit embeds within a larger horizontal composite. This reflects the sensitivities to the contiguity of panel borders set out in the first two preference rules. Satisfying the length of the vertical boundaries allows the broader horizontal structure to be completed only through the addition of the third panel. Using a Z-path would flout this by finishing the horizontal structure of panels A and C first, as in Figure 8B. This completion leaves the remaining panel as an unnecessary gap in the Assemblage process. If B is left to an additional horizontal structure, that grouping “runs through” the already existing C panel. This adds redundancy, and forces the reader to jump over the already read panel. Meanwhile, following the Z-path in blockage scenarios flouts all of the Assemblage constraints. In part, this is the challenge created by the Steranko page in Figure 1 – no matter whether the reader follows blockage or the Z-path, the layout forces the reader to jump over a panel because no clean segments can be grouped. (Despite this, analysis of participants’ preferences in the experimental Steranko page did show consistent reading order strategies. See the online supplement for this analysis.)

**Figure 9:**
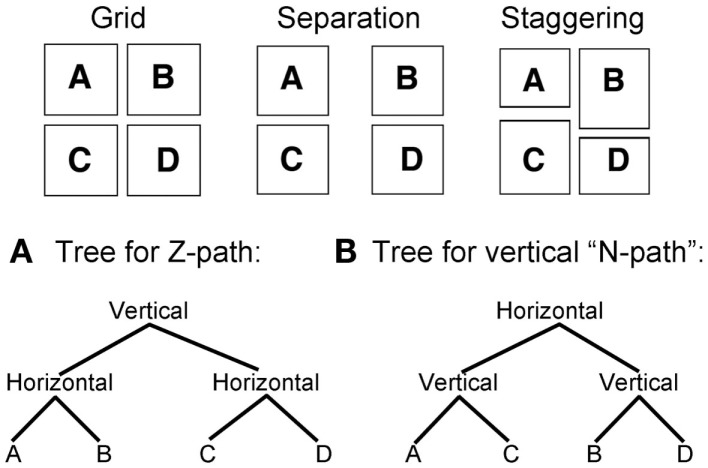
**Tree structures for reading orders of various grids using (A) a strategy following the Z-path, or (B) a strategy going against the Z-path (for a vertical “N-path”)**.

Similarly, using this tree structure formalism, the Z-path would be represented as in Figure 9A. Under an overarching vertical structure, horizontal segments concatenate various panels in rows. This is the way to represent successive reading of multiple rows moving downward. Any number of horizontal nodes can be added into this Z-path, as well as any number of panels within each horizontal node. A general computational rule reflecting the Z-path preference rules could be stated as (with asterisks notating repetition):

Z - path rule: [Vertical [Horizontal * ⌊Unit*⌋]]

This constituency rule states that within an overarching vertical unit, an unlimited number of horizontal segments can embed, into which an unlimited number of units can be placed. This is essentially the default rule for Western style reading of text. Altering it to suit the vertical motions of Japanese or Chinese would merely require the flipping of the horizontal and vertical labels (as in Figure 9B). An associated constraint would then specify the direction of motion, moving right, left, up, and/or down.

These groupings further explain why separation could statistically depart from the Z-path yet be numerically chosen less than the Z-path. Assemblage works for both routes in Figures 9A,B, allowing for composite structures to build cleanly using smooth motions, without leaving gaps. As depicted in Figure 9A, the tree structure for separation greatly resembles that of the Z-path, only inverting the vertical and horizontal nodes. This same tree would appear for staggering, which showed a trend toward departing from the Z-path. Like separation, staggering forces the reader to decide which boundary to complete first: horizontal or vertical. Both strategies of the Z-path and vertical reading allow Assemblage to be completed without gaps, using a smooth motion. However, the engagement with the preference rules determines which path will ultimately be chosen.

**Figure 10:**
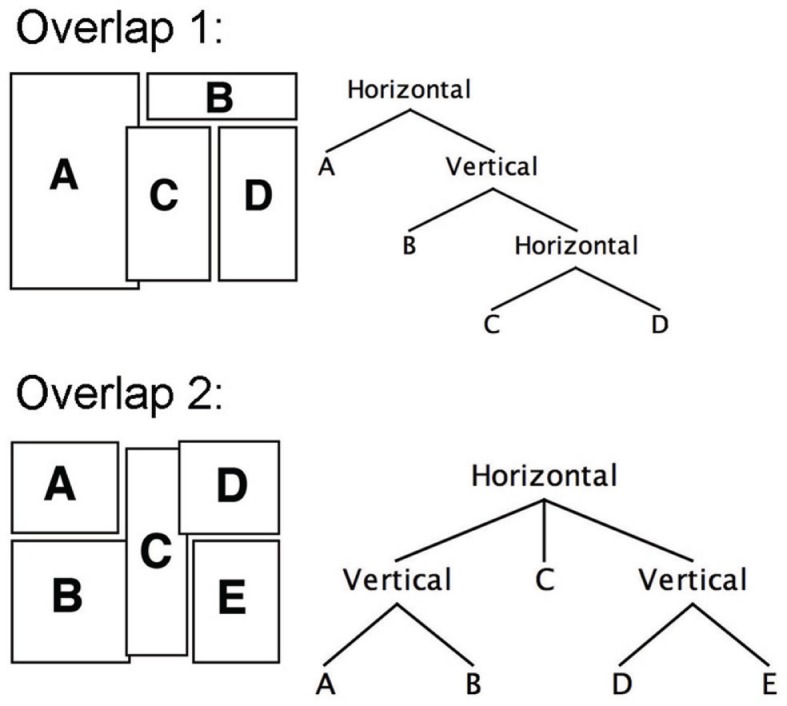
**Tree structures for overlapping examples**.

This search for adequate grouping strategies also accounts for the significant differences in the items analysis between overlap conditions. While the overlap in Figure 3K allowed for Assemblage through the overlapping panel, Figure 3F allowed Assemblage through the Z-path while going against the overlapping panels. In both cases, participants preferred to follow Assemblage, regardless of overlap or Z-path. For example, in Figure 3F, the dominantly chosen path went against overlap. The dominant reading path for this segment fulfilled ECSPR 1 and uppermost edge first, then accounted for the internal edges – wholly discarding the proximity of the overlap. Interestingly, the resulting structure is a left branching tree, since it continually embeds further into each subsequent layer, as depicted in Figure 10. The cluster in Figure 3K primarily followed the overlap path. In this case, the vertical border (A–B) allowed the first completion, which just so happened to follow the overlap through the blockage. Through analysis of these stimuli at least, overlap has little effect on reading strategy, which relies more on the preference rules and Assemblage. This is surprising since it contrasts the logical associations that would be made through both the Z-path and the Gestalt principles of proximity.

The left branching aspect of Figure 3F highlights an important aspect to the ECS system: recursion. Any horizontal structure can embed a vertical structure, and vice-versa, which means that embedding can go on infinitely (theoretically speaking). Note also that a node of one type cannot embed a node of the same type.

These rules guide the entire embedding process for ECS, with a few exceptions like inset panels. They are emblematic, in that for a 2D space, the grouping rules concatenate elements along *X* and *Y* dimensions. These grouping rules are also idealizations, in that not all constituents cleanly group rectangular panels. Truly, the same rules would apply to panels diagonally arranged or with angled gutters for the overarching grouping patterns, further constrained by the ECSPR.

### Descriptive tree structures

The tree structure approach to ECS can also offer a way to describe the structure of full comic pages. In this case, these “descriptive” trees would articulate the underlying structure used by an author to create particular comic pages, and presumably decoded by a reader in their navigation. As a descriptive tool, this approach has several benefits. For example, this notation can be used in conjunction with the trees created by narrative structures (e.g., Cohn, 2013) to show the interfacing between a sequence’s content and its layout. Such descriptions could also benefit efforts to characterize the differences between page layouts within and between comics, providing a quantitative method to assess the structures of ECS cross-culturally through corpus analyses (which has yet to be undertaken).

**Figure 11:**
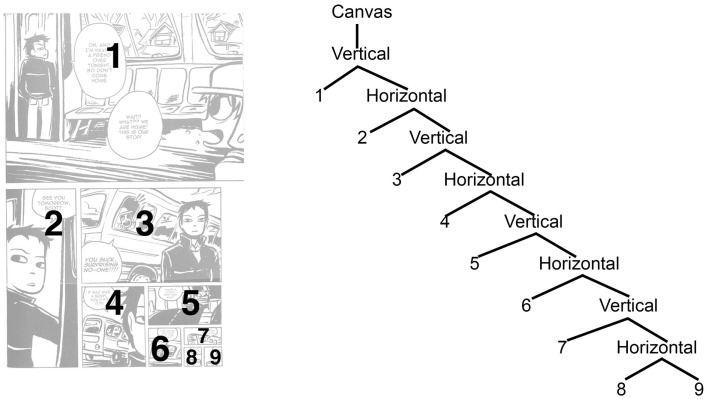
**Page from *Scott Pilgrim vs. The World* with recursive embedding**. *Scott Pilgrim vs. The World* is © 2005 Bryan Lee O’Malley.

For full pages, a topmost node of “Canvas” can be added to the overall trees – taken here to mean the maximal space that the ECS covers. This could be a single page, a two page spread, or the overall space of a website, wall, piece of pottery, etc. A few example pages from various comics can illustrate this formalism. Figure 11 excellently demonstrates recursive embedding in a comic page layout from the comic *Scott Pilgrim* vs. *The World*, since each panel is successively embedded within either a horizontal or vertical node. This technique results in a layout where each panel gets progressively smaller, coinciding with the narrative of one character fading into the distance riding on a bus. In effect, the recursive structure of the layout facilitates the sense that the character recedes into the distance away from the reader.

**Figure 12:**
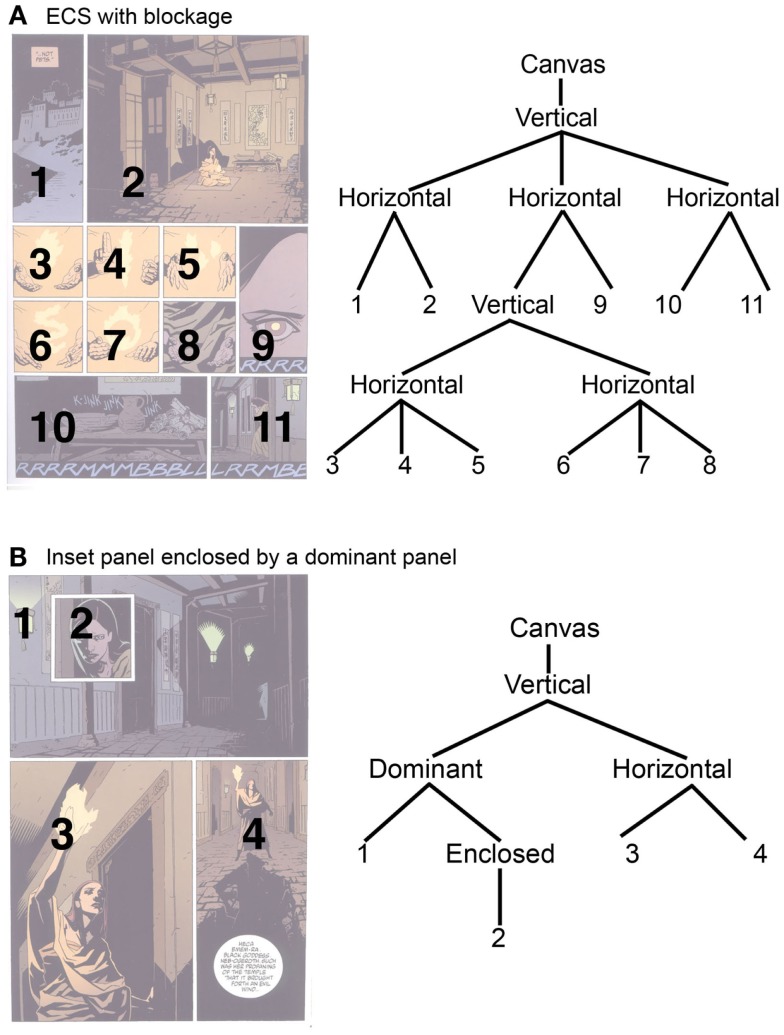
**Pages from Mike Mignola’s *B.P.R.D*. with (A) blockage and (B) an inset panel**. © 2003 Mike Mignola.

Other good examples come from Mike Mignola’s *B.P.R.D*., as in Figure 12A. This canvas is divided into three horizontal streams connected through an overarching vertical node. Blockage splits the second horizontal node into two more horizontal sub-streams (345, 678) before reaching panel 9. Note that as long as panel 8 immediately precedes panel 9, the content of the panels within these subordinate rows could be read as vertical columns (with the broader row encapsulating three vertical nodes). However, based on the rules of Assemblage, they would be read horizontally, since no guiding force like the length of panel 9 dictates a vertical order. Essentially, the rows belonging to panels 5 and 8 engage in the blockage condition additively for their respective rows, since they form that interaction with panel 9.

Figure 12B illustrates the next page of the same book. This page is relatively simpler, with only four panels. The whole page divides into two sections bound through an overarching vertical node. The bottom segment is a horizontal row, though the top half features an inset panel enclosed within another. In tree form, the dominant panel embeds the enclosed panels inside it. Note that the enclosed node could feature multiple panels within it, and also include another “enclosed” node for an inset within an inset (indefinitely, since this too becomes recursive).

### Conclusion

This study examined participants’ intuitions for navigating through the panels of comic pages. We found that navigation of comic pages follows strategies that extend beyond the Z-path used to read written text and common Gestalt groupings like proximity. “Assemblage” constraints and preference rules comprise a system for navigating an ECS, that work toward the building of hierarchic constituent structures. There are several avenues of research that can follow this work.

First, as discussed, future studies can further investigate the precise balancing of probabilistic weights that go into preference rules and Assemblage constraints. This approach has provided an initial sketch for a previously unaddressed question, and has introduced both basic notions about the structure of page layouts (blockage, staggering, separation, etc.) and principles for how they are navigated (Assemblage, preference rules, constituent structure). Future work can clarify, enhance, or revise the principles of this model with more precise manipulation of these basic structural constructs.

Second, while this structure is separate from meaning – evident since consistent results emerged even with empty panels – this does not mean that ECS and narrative in panels cannot or do not interact. Having shown that readers *do* have a system for navigating layouts outside content, it is an open question as to what happens when the content defies these principles. Do readers face difficulties when layout and narrative mismatch? Does the content override the ECSs? Does this create costs in processing? There should be little doubt that the content of panels and a sequence can further constrain the navigation of readers through a layout, yet the precise manner by which this is done remains an open question. In all likelihood, an additional set of constraints must weigh factors like color, panel composition, character’s positioning and eye-gaze, and breaking the borders of panels. Experiments using panels with ambiguous narrative content that allows for more than one felicitous reading order could likely manipulate such traits in clever ways.

Finally, these results allow for future research to connect with work that has been done on the navigation of other media and writing systems. Do comic readers – who have preferences for explicit types of reading orders – engage with newspapers and websites using different strategies than the general scanning found in previous studies? How do the ECSPR interact with more general rules of scanning and engagement used in other media? Does the varied reading order created by reading comics change the biases created by the direction of writing systems with regard to depicting temporal relationships (Chan and Bergen, 2005; Tversky et al., 1991), assigning semantic agency to objects (Dobel et al., 2007; Maass and Russo, 2003), and other perceptual tasks? This research can open the door to further research connecting the navigation of media across several domains.

## Conflict of Interest Statement

The authors declare that the research was conducted in the absence of any commercial or financial relationships that could be construed as a potential conflict of interest.

## Graphic References

All images are created and copyright © 2013 Neil Cohn, except those cited throughout the text. Cited images are copyright their respective owners and used purely for analytical, critical and scholarly purposes.

Mignola, M., and Sook, R. (2003). *Mike Mignola’s B.P.R.D.: Hollow Earth and Other Stories*. Milwaukie, OR: Dark Horse Comics.

O’Malley, B. L. (2005). *Scott Pilgrim vs. The World*. Portland, OR: Oni Press.

Steranko, J. (2002). *Marvel Visionaries: Steranko*. New York, NY: Marvel Comics.
