# Erratum: Navigating comics: an empirical and theoretical approach to strategies of reading comic page layouts

**DOI:** 10.3389/fpsyg.2014.00015

**Published:** 2014-01-24

**Authors:** Neil Cohn

**Affiliations:** Center for Research in Language, University of California San DiegoLa Jolla, CA, USA

**Keywords:** comics, page layout, spatial representation, reading order, writing systems

This General Commentary provides a corrected version of the caption for **Figure 2**. The text should read:

**(A)** Canonical grid layout stereotypically read in a “Z-path.” **(B)** Layout where a vertical panel “blocks” the creation of a row of panels. **(C)** Layout where panels are separated by a wide space. **(D)** Layout where panels overlap each other. **(E)** Layout where panels are staggered to no longer retain a contiguous gutter.

In addition, the link to the supplementary analysis of the Steranko page has been changed to: http://www.visuallanguagelab.com/P/ECS_Supplement.pdf

